# Metabotropic glutamate receptor 5 regulates synaptic plasticity in a chronic migraine rat model through the PKC/NR2B signal

**DOI:** 10.1186/s10194-020-01206-2

**Published:** 2020-12-04

**Authors:** Yingying Niu, Xiaoxu Zeng, Lilin Zhao, Yang Zhou, Guangcheng Qin, Dunke Zhang, Qingqing Fu, Jiying Zhou, Lixue Chen

**Affiliations:** 1 Laboratory Research Center, The First Affiliated Hospital of Chongqing Medical University, Chongqing, China; 2 Department of Stomatology, The First Affiliated Hospital of Chongqing Medical University, Chongqing, China; 3 Department of Radiology, The First Affiliated Hospital of Chongqing Medical University, Chongqing, China; 4 Department of Neurology, The First Affiliated Hospital of Chongqing Medical University, Chongqing, China

**Keywords:** Migraine, Synaptic plasticity, mGluR5, NR2B, PKC

## Abstract

**Background:**

The mechanism of chronic migraine (CM) is complex, central sensitization is considered as one of the pathological mechanism. Synaptic plasticity is the basis of central sensitization. Metabotropic glutamate receptor 5 (mGluR5) plays a vital role in the synaptic plasticity of the central nervous system. However, whether mGluR5 can promote the central sensitization by regulating synaptic plasticity in CM is unknown.

**Methods:**

Male Wistar rats were used to establish a CM rat model, and the expression of mGluR5 mRNA and protein were detected by qRT-PCR and western blot. The allodynia was assessed by mechanical and thermal thresholds, and central sensitization was assessed by expression of the phosphorylation of cyclic adenosine monophosphate (cAMP) response element-binding protein (CREB) at Serine 133(pCREB-S133) and c-Fos. The synaptic-associated protein postsynaptic density protein 95 (PSD), synaptophysin (Syp), and synaptophysin-1(Syt-1), synaptic ultrastructure, and dendritic spines were detected to explore synaptic plasticity. The expression of PKC, total NR2B(tNR2B), and phosphorylation of NR2B at Tyr1472(pNR2B-Y1472) were detected by western blot.

**Results:**

We found that the expression of mGluR5 was upregulated in CM rats. Downregulated the mGluR5 with MPEP alleviated the allodynia and reduced the expression of CGRP, pCREB-S133, c-Fos, PSD, Syp and Syt-1 and synaptic transmission. Moreover, the administration of MPEP inhibited the upregulation of PKC and pNR2B-Y1472.

**Conclusions:**

These results indicate that mGluR5 contributes to central sensitization by regulating synaptic plasticity in CM through the PKC/NR2B signal, which suggests that mGluR5 may be a potential therapeutic candidate for CM.

## Introduction

Migraine is one of the most prevalent neurological diseases, which is classified as episodic migraine (EM) and CM [1]. Every year, 2.5–3.0% of patients with EM progress to CM [2]. CM refers to at least 8 migraine-like attacks per month [3], seriously affecting the life quality of patients [4]. Therefore, it is urgent to deeply understand the molecular mechanism of CM to provide support for clinical treatment.

Central sensitization is considered to be the main pathogenesis of CM [5]. Central sensitization mainly occurs in the secondary neurons of the trigeminal nucleus caudate (TNC) [6]. The enhancement of synaptic plasticity is the basis of central sensitization in CM. Our previous studies have shown that the ionotropic glutamate N-methyl-D-aspartate (NMDA) receptor regulates the synaptic plasticity of CM model rats and participates in central sensitization [7], indicating that glutamate and its receptors play a role in the synaptic plasticity of CM. Whether metabotropic glutamate receptors can regulate the synaptic plasticity of CM is of interest to us.

MGluR5, a subtype of type I mGluRs, is a G protein-coupled receptor (GPCR), associated with a variety of nervous system diseases, including pain, Alzheimer’s disease [8], and addiction [9]. Previous studies have shown that mGluR5 regulates the induction and maintenance of LTP in the hippocampal CA1 region of rats. Besides, proper inhibition of mGluR5 regulates glutamate related overactivity and postsynaptic excitatory synaptic transmission in Parkinson’s disease [10, 11]. All these indicate that mGluR5 plays an important role in the regulation of synaptic plasticity. The activation of mGluR5 stimulates phospholipase C β-related pathways to produce inositol-1,4,5-triphosphate (IP3) and diacylglycerol. IP3 activates calcium-dependent ion channels and releases calcium from cellular stores; intracellular calcium stimulates protein kinase C (PKC) and its related downstream signaling pathways [12–14]. PKC phosphorylates transcription factor CREB through NMDAR/calmodulin-dependent protein kinaseII (CaMKII) sensitive signal pathway and participates in the pathophysiological process of synaptic plasticity in the central nervous system [15, 16]. Therefore, we proposed a scientific hypothesis that mGluR5 participates in the central sensitization by regulating synaptic plasticity through PKC/NR2B signal in CM.

Based on the above hypothesis, we investigated the role of mGluR5 in CM. We established a CM rat model by infusing inflammatory soup (IS) into the dura mater and detected a significant increase of the expression of mGluR5 in TNC. Intracerebroventricular administration of mGluR5 antagonist MPEP reduced synaptic plasticity through PKC/NR2B signal and alleviated central sensitization in CM rats. Thus, mGluR5 might be a therapeutic target for the CM.

## Meterials and Metholds

### Animals

Male Wistar rats weighing 250-300 g (specific pathogen-free; certificate no. SCXK [LIAO]2015–0001; Liaoning, China) were obtained from Liaoning Chang Sheng Biotechnology Co., Ltd. (Benxi, Liaoning, China). The rats were maintained on a 12–12 light/dark cycle, temperature (23 °C ± 2 °C), and humidity (50% ± 10%) laboratory condition with enough food and water. All animal procedures followed the National Institutes of Health Guide for the Care and Use of Laboratory Animals (NIH Publications No. 80–23, revised 1996). After a week’s acclimatization, rats were randomly assigned to each experimental group. The study was approved by the Ethics Committee of the Department of Medical Research (First Affiliated Hospital of Chongqing Medical University).

### Establishment of the CM model

After fasting for 8 h and general anesthesia, the rats were placed in a stereotactic apparatus (ST-51603; Stoelting Co., Chicago, IL, USA) with shaved. The surgical blade sterilized by alcohol was used to cut along the midline of the rat’s head to expose the skull. A 1-mm-diameter craniotomy in the right frontal bone (− 1.0 mm rear from the bregma, + 1.5 mm lateral to the bregma) was performed using the skull drill without damaging the dura mater, and a sterile stainless-steel cannula was fixed to the bone window using dental fixation acrylic. A paired cap was used to seal the cannula to keep it unobstructed and an iodophor was used to disinfect the region of surgical. Then, the rats were maintained on an electric heating blanket until they awoke from anesthesia and placed back to their cages. The rats were allowed to recover for one week before dural infusions until their mechanical and thermal thresholds returned to preoperative levels. The rats were infused with 2 μL IS for 7 days to dura to establish a CM rat model as previously reported, and the IS was composed of 1 mM bradykinin, 1 mM histamine, 1 mM serotonin, and 0.1 mM prostaglandin E2 (Sigma-Aldrich, St. Louis, MO, USA) in PBS (Ph 7.4). The vehicle control was 2 μL PBS. The process of infusion lasted 10 min to make sure that the IS or PBS spread to the dura.

### Drug delivery

To understand the role of mGluR5 in CM, the mGluR5 antagonist 6-methyl-2-(phenylethynyl) pyridine hydrochloride (MPEP; 0.2 μg, 2 μg, 5 μg/5 μL; Sigma-Aldrich, St. Louis, MO, USA) was administered into the lateral ventricle through a sterile stainless-steel cannula. MPEP was dissolved in dimethyl sulfoxide (DMSO) solution, which was used as vehicle control. The rats were randomly divided into the following groups: (1) sham group,(2) CM group,(3)CM + DMSO group,(4)CM + MPEP-LD group(0.2 μg/5 μL), (5)CM + MPEP-HD group(2 μg/5 μL), (6)CM + MPEP-HD group(5 μg/5 μL). The sample size of each group was shown in Table 1.

**Table 1:** Animal numbers in each group

Experimental group	Behavioral tests	qRT-PCR	WB	IF	TEM	Golgi-Cox
Sham	6^a^	4	4	4	4	4
CM	6^a^	4	4	4	4	4
CM + DMSO	6^a^	–	4	4	4	4
CM + MPEP(0.2 μg)	6	–	–	–		–
CM + MPEP(2 μg)	6^a^	–	4	4	4	4
CM + MPEP(5 μg)	6	–	–	–	–	–

^a^Indicates shared with other experiments,do not count; − tests were not done

### Behavioral tests

Behavioral tests of rats included the measurements of mechanical thresholds and thermal thresholds. Each rat’s thresholds were measured three times at each time point with an interval of one minute. As previously described [7], the electronic von Frey instrument (Electrovonfrey, 2391, IITC Inc., Woodland Hills, CA, USA) was used to detect the mechanical thresholds of the hind paw. After 20–30 min of acclimation, the pressure probe tip was applied to the detected region of the rat, starting from a small force value and gradually increasing until the rapid lifting of the hind paw from the metal grid. The plantar test instrument (model PL-200, IITC, Taimeng, Chengdu, China) was used to measure the thermal thresholds as previously described [17]. The rats were placed in a transparent cage for acclimation for 30 min, and then infrared radiation (intensity, 20%) was applied to the surface of the hind paw of the rat until the hind paw of the rat was raised, then, the withdrawal latency was automatically recorded by the instrument.

### Quantitative real-time reverse transcriptase polymerase chain reaction (qRT-PCR)

After the establishment of the CM rat model, the rats were sacrificed, and the TNC tissue was quickly separated and stored in liquid nitrogen for qRT-PCR. The RNAiso Plus reagent (TaKaRa, Dalian, China) was used to extract total RNA from TNC tissue, and the RNA concentration was measure by a NanoDrop spectrophotometer (Thermo, USA). Then, cDNA was synthesized by PrimeScript TM RT Reagent Kit (TaKaRa). Finally, the qRT-PCR was performed on a CFX96 Touch thermocycler (Bio-Rad, USA) using SYBR®Premix Ex TaqTM II (TaKaRa, Dalian, China). Gene expression was expressed as the target/reference ratio by a post-PCR data analysis software program, and differential analysis was performed by the 2 − ΔΔCT method. The primer sequences were used as follows:mGluR5(Forward Primer):5′-GAAAGGCCAAATAAAGGTGATCCG-3′,mGluR5(Reverse Primer: 5′-GCGAAGATACTGGACTGGGA-3′); GAPDH (Forward Primer):5′-ATGACTCTACCCACGGCAAGC-3′, GAPDH (Reverse Primer: 5′-GGATGCAGGGATGATGTTCT-3′).

### Western blot analysis

The TNC tissue was homogenized in radioimmunoprecipitation assay (RIPA) lysis buffer (Beyotime, Shanghai, China) with phenylmethylsulfonyl fluoride (PMSF, Beyotime, Shanghai, China) and protein concentration was measured by bicinchoninic acid (BCA) protein assay kit (Beyotime, Shanghai, China). The proteins were loaded on SDS-PAGE for electrophoresis and transferred to PVDF membranes. After blocking in Tris-buffered saline containing Tween 20 (TBST) containing 5% dried not-fat milk at room temperature for 2 h, the membranes were incubated with primary antibody at 4 °C overnight. The next day, the membranes were incubated with horseradish peroxidase-conjugated secondary antibodies at room temperature for 1 h and visualized using a BeyoECL Plus kit (Beyotime, Shanghai, China). The imaging system (Fusion, Germany) was used to analyze the immunoblots. The specific information for each antibody used for western blot is shown in Table 2.

**Table 2:** Antibodies used in Western blot analysis and immunofluorescent staining

Antibody	Manufacturer	Dilution
For Western blot analysis		
β-actin	Proteintech, USA	1:3000
GAPDH	Abways Technology, China	1:5000
mGluR5	Milipore,USA	1:3000
CGRP	Abcam, UK	1:3000
PSD95	Abcam, UK	1:1000
Synaptophysin	Abcam, UK	1:5000
PKC	Abcam, UK	1:3000
NR2B	Proteintech, USA	1:1000
p-NR2B-Y1472	Bioss, China	1:500
CREB	Abcam, UK	1:1000
p-CREB-S133	Abcam, UK	1:3000
c-Fos	Novus Biologicals, USA	1:3000
Anti-rabbit IgG (HRP)	Bioss, China	1:9000
Anti-mouse IgG (HRP)	Zhongshan Golden Bridge Bio, China	1:5000
For Immunofluorescent Staining		
CGRP	Santa Cruz, USA	1:100
Synaptotagmin-1	Bioss, China	1:200
c-Fos	Synaptic Systems, Germany	1:800
Cy3-conjugated goat anti-rabbit IgG	Beyotime, China	1:500
Alexa Fluor 488 goat anti-rabbit IgG	Beyotime, China	1:500
Alexa Fluor 488 goat anti-mouse IgG	Beyotime, China	1:500

### Immunofluorescence staining

After general anesthesia, the rats were perfused transcardially with 4% paraformaldehyde, and the TNC region was immediately separated. The separated tissues were postfixed with 4% paraformaldehyde at 4 °C for 12 h and immersed in 20% and 30% sucrose solution to dehydrate. The 10 μm sections were cut by a cryostat (Leica) and were blocked with 10% normal goat serum (Boster, Wuhan, China). Then, the sections were incubated with the primary antibody at 4 °C overnight. The next day, the sections were incubated with the secondary antibody at 37 °C for 90 min and 4′,6-diamidino-2-phenylindole (DAPI) for 10 min at 37 °C. The 50% glycerol was used to seal the sections and the confocal laser scanning fluorescence microscope (ZEISS, Germany) was used to visualize the sections. The specific information for each antibody used for immunofluorescence staining is shown in Table 2.

### Transmission electron microscopy

After general anesthesia, the rats were perfused transcardially with 2.5% glutaraldehyde, and the TNC was separated and immersed in 4% glutaraldehyde at 4 °C for 24 h. Then, the TNC tissues were cut into 1-mm^3^ pieces and sent to Chongqing Medical University for post fixing, embedding, sectioning, and staining. The images of synapses were obtained by JEM-1400 PLUS transmission electron microscope (TEM) and analyzed with Image Pro Plus. The thickness of the postsynaptic density (PSD), the length of the synaptic activity zone, and the synaptic interface curvature was determined as previously reported [18, 19]. (*n* = 4 rats per group, 5 images per animal).

### Golgi-cox staining

An FD Rapid Golgi Stain Kit™ (FD NeuroTechnologies-Columbia, MD, USA) was used for tissue preparation and staining. The fresh TNC tissue was isolated and washed with double distilled water to remove the blood from the surface. Then, the tissue was immersed in a premixed mixture (solution A and solution B 1:1, the solution was changed once after 24 h) for two weeks and transferred into solution C (the solution was changed once after 24 h) for 3 days at room temperature in the dark. A series of 150-μm sections were cut with a vibratome (Leica VT 1200S, Japan) and stained according to the manufacturer’s instructions. Finally, the slides were sealed with Permount™ Mounting Medium (Fisher Scientific Co, Waltham, MA, USA). The dendritic spines were imaged with a Zeiss microscope (Axio Imager A2) [20].

### Statistical analysis

Data are presented as the mean ± SD. SPSS 23 software was used for statistical analysis, and GraphPad Prism 8 was used to generate graphs. Behavioral data were analyzed by two-way analysis of variance (ANOVA) followed by a Bonferroni post hoc test. The differences between the two groups were assessed by two independent sample t-tests. Multiple comparisons were analyzed by one-way ANOVA and a Bonferroni post hoc test. The values of *p* < 0.05 were considered to be statistically significant.

## Results

### Reduction of pain thresholds and elevation of CGRP after the induction of CM in rats

**Fig. 1:**
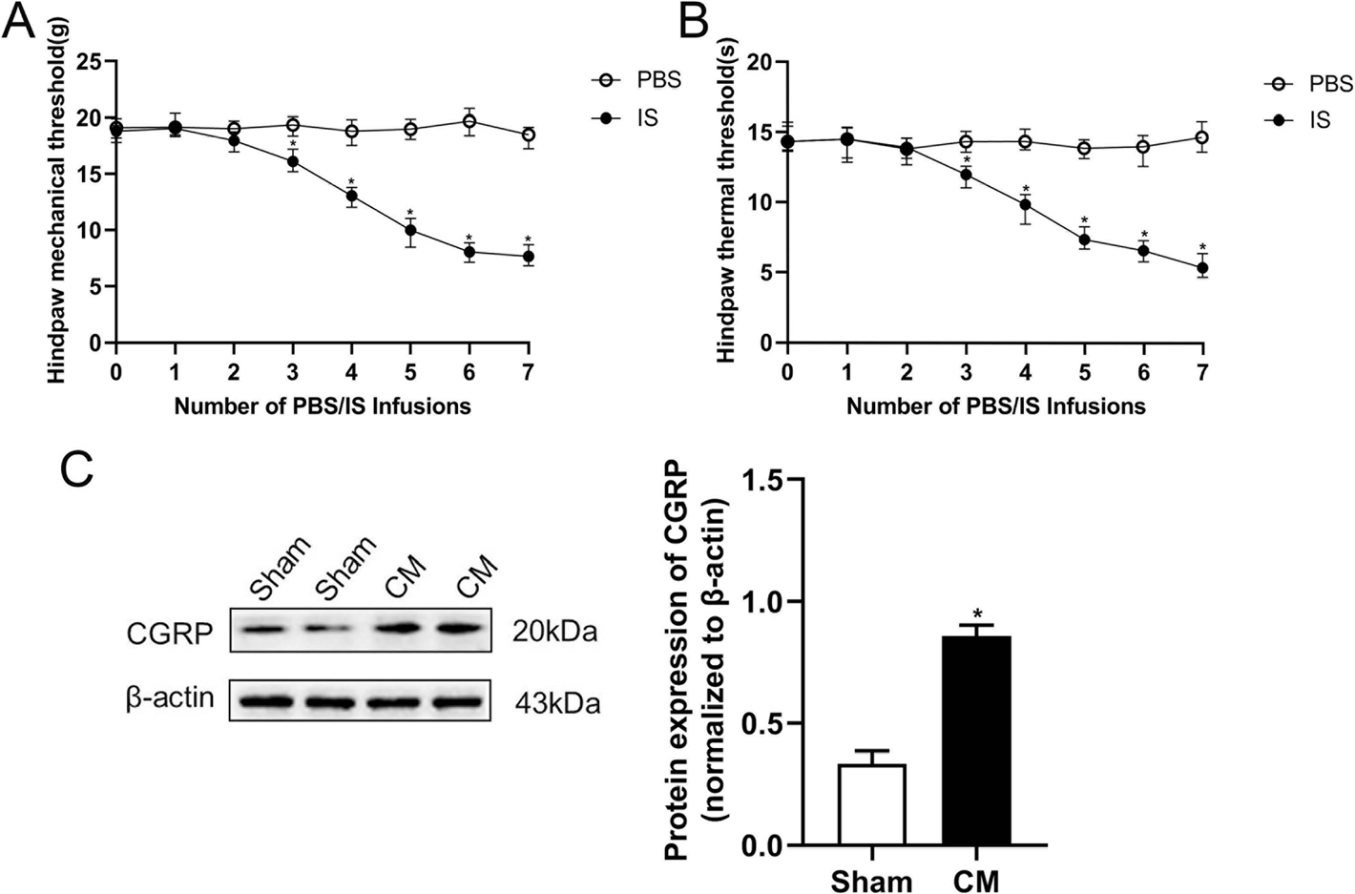
Repeated IS infusions induced mechanical allodynia and thermal allodynia and upregulated the expression of CGRP in the rats. **a** The mechanical pain thresholds of the hind paw during the infusion of PBS/IS. **b** The thermal pain thresholds of the hind paw during the infusion of PBS/IS. **c** The protein expression of CGRP. (*n* = 6 in each group; *p < 0.05 compared with the sham group)

We established a rat model of CM by continuous infusion of IS into the dura mater for seven days and evaluated the success of the model by the mechanical pain thresholds, thermal pain thresholds, and calcitonin gene-related peptide (CGRP) expression level. The PBS group was used as the control group. There was a significant decrease in the mechanical pain thresholds and thermal pain thresholds of hind paw of the CM group rats from the third day after the infusion of IS (Fig. 1a,b). In addition, CGRP is considered to be closely related to the pathological mechanism of CM, and its expression level is used as a key index to evaluate the success of the model [21], so we also detected the expression of CGRP, and we found that repeated dural infusion of IS significantly increased the expression of CGRP (Fig. 1c). The above results show that we establish a successful and reliable rat model of CM, which can be used in follow-up experiments.

### Upregulation of mGluR5 expression in the TNC of CM rats

**Fig. 2:**
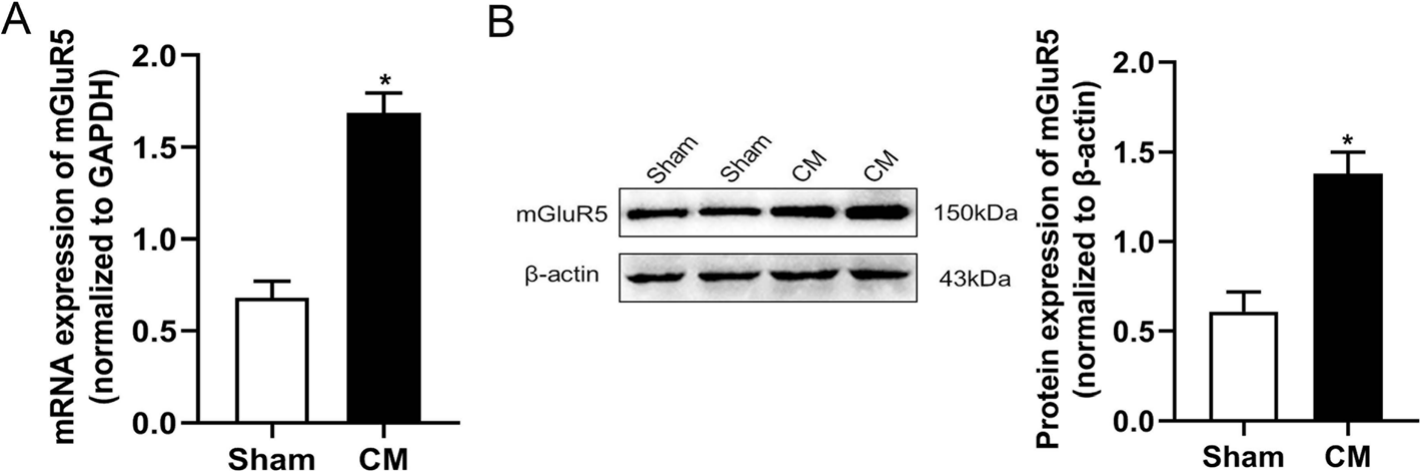
Repeated IS infusions upregulated the mRNA and protein expression of mGluR5 in the TNC of rats. **a** The mRNA expression of mGluR5. **b** The protein expression of mGluR5. (*n* = 4 in each group; *p < 0.05 compared with the sham group)

After the successful establishment of the CM rat model, qRT-PCR and western blot analysis techniques were used to measure the expression of mGluR5 in TNC. The results of qRT-PCR showed that compared with the sham group, the mRNA level of mGluR5 in the CM group rats was significantly increased (Fig. 2a). Consistent with the results of qRT-PCR, the protein level of mGluR5 was significantly higher than that of the sham group (Fig. 2b). The results show that the expression of mGluR5 is upregulated in CM, which suggests that the mGluR5 signal may play an important role in CM.

### Downregulation of mGluR5 attenuated the hyperalgesia and reduced the expression of CGRP in CM rats

**Fig. 3:**
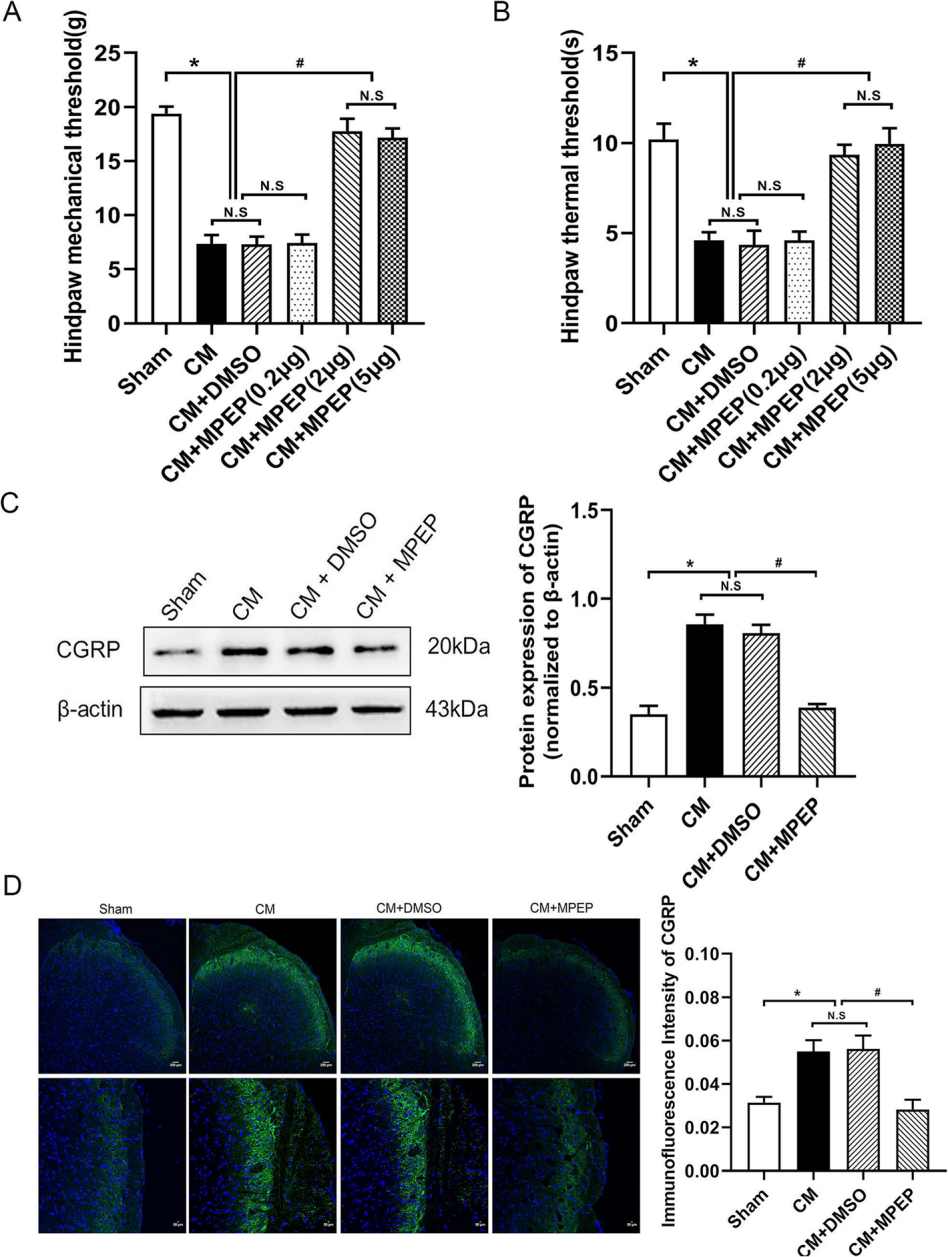
The effect of MPEP on mechanical allodynia, thermal allodynia, and the expression of CGRP. **a-b** Compared with the sham group, pain thresholds significantly decreased in the CM group. There were no significant differences between the CM and CM + DMSO groups. Compared with the CM and CM + DMSO groups, 0.2 μg MPEP did not effect, while 2 μg and 5 μg MPEP all increased the pain thresholds. There were no significant differences between the CM + MPEP(2 μg) and CM + MPEP(5 μg) groups. **a** The mechanical pain thresholds of the hind paw in different groups. **b** The thermal pain thresholds of the hind paw in different groups. **c-d** Compared with the sham group, the expression of CGRP increased in the CM group. There were no significant differences between the CM and CM + DMSO groups. The administration of MPEP significantly downregulated the expression of CGRP. **c** The protein expression of CGRP. **d** Immunoreactivity for CGRP. (*n* = 4 in each group, scale bar = 200 μm or scale bar = 50 μm; *p < 0.05 compared with the sham group; ^#^*p* < 0.05 compared with the CM + DMSO group; N. S:*p* > 0.05 between two groups)

We have confirmed that mGluR5 was significantly upregulated in CM rats. Therefore, we administered mGluR5 antagonist MPEP to the lateral ventricle to explore the effect of mGluR5 on hyperalgesia and CGRP. Compared with the sham group, the mechanical thresholds (Fig. 3a) and the thermal thresholds (Fig. 3b) of the CM group were significantly decreased. The administration of DMSO alone into the lateral ventricle did not affect the pain thresholds. The administration of medium-dose(2 μg/5 μL) or high-dose(5 μg/5 μL) MPEP increased the mechanical pain thresholds and thermal pain thresholds of the CM rats, while the low-dose(0.2 μg/5 μL) had no obvious effect, so we choose medium-dose MPEP (2 μg/5 μL) for subsequent experiments. CGRP is expressed in trigeminal ganglion neurons and participates in the regulation of trigeminal nerve innervation and nociceptive transmission in CM. Consistent with the pain thresholds results, the western blot (Fig. 3c) and immunofluorescence (Fig. 3d) results showed that the administration of MPEP (2 μg/5 μL) reduced the high expression of CGRP in the CM rats. These results indicate that mGluR5 is involved in CM.

### Downregulation of mGluR5 reduced expression of synaptic associated proteins

**Fig. 4:**
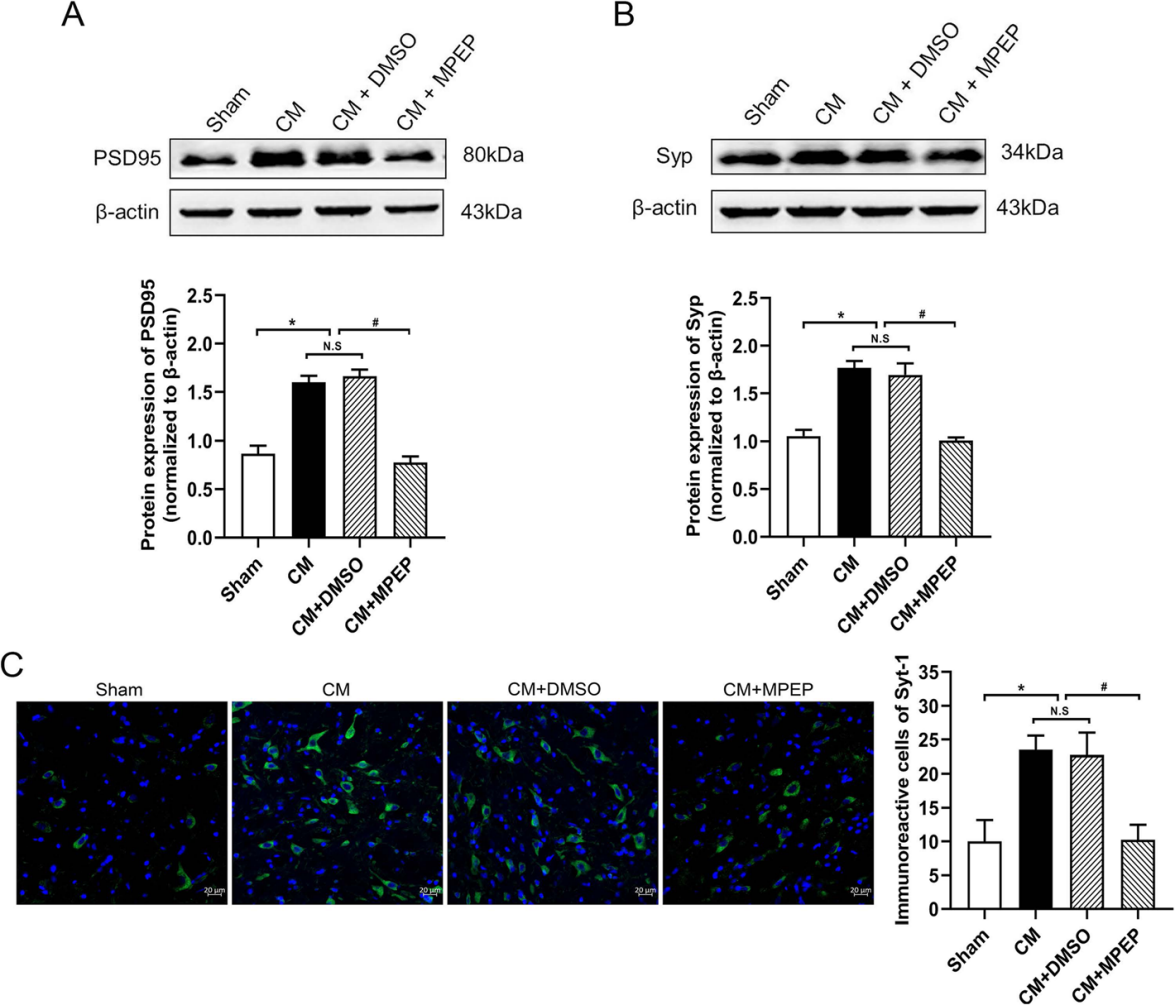
The effect of MPEP on synaptic associated proteins. Compared with the sham group, the expression of PSD95, Syp, and Syt-1 upregulated in the CM group. There were no significant differences between the CM and CM + DMSO groups. The administration of MPEP significantly decreased their expression. **a** The protein expression of PSD95. **b** The protein expression of Syp. **c** Immunoreactivity for Syt-1. (*n* = 4 in each group,scale bar = 20 μm; *p < 0.05 compared with the sham group; ^#^p < 0.05 compared with the CM + DMSO group; N. S:p > 0.05 between two groups)

We detected synaptic associated proteins after administration of mGluR5 antagonist MPEP to explore whether mGluR5 can regulate the synaptic plasticity of CM. Western blot was used to detect PSD and Syp, immunofluorescence was used to detect Syt-1. Compared with the sham group, the expression of PSD (Fig. 4a) and Syp (Fig. 4b) protein and the number of Syt-1-positive cells (Fig. 4c) in the CM group were significantly increased. There was no significant difference between the CM and CM + DMSO groups. The administration of MPEP decreased the expression of PSD and Syp proteins and the number of Syt-1-positive cells. These results suggest that mGluR5 may regulate the synaptic plasticity of CM.

### MGluR5 regulated the ultrastructure of synapses

**Fig. 5:**
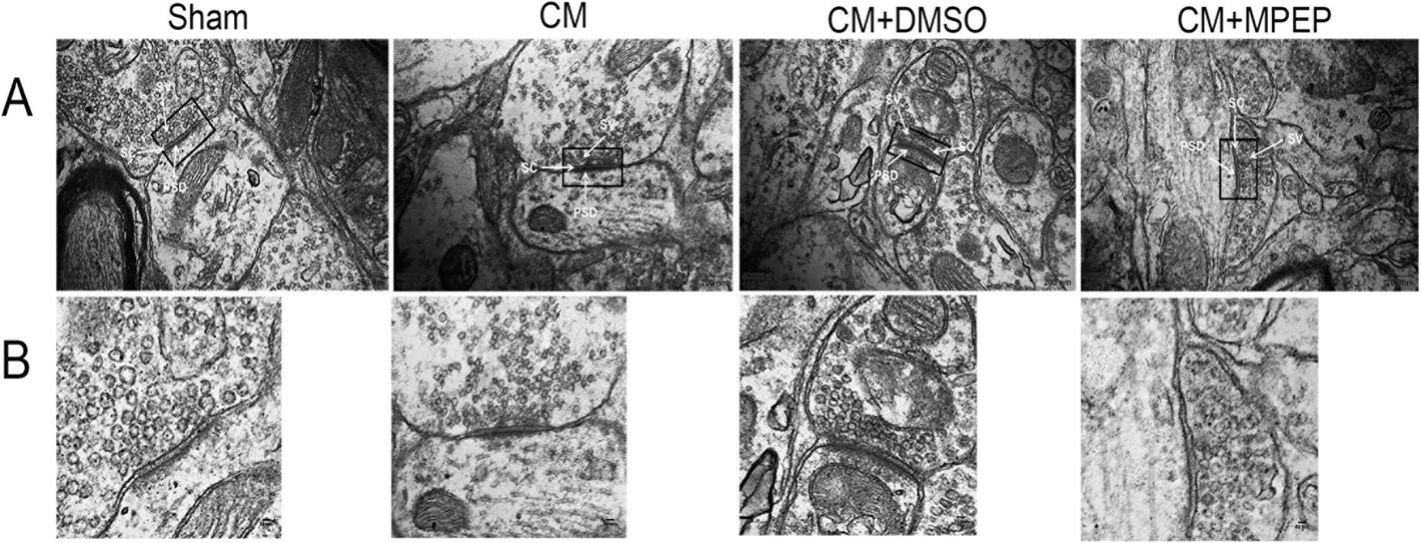
Changes of synaptic ultrastructure. **a** Synaptic ultrastructure in the different groups. (Scale bars = 200 nm) **b** Magnification of the black rectangle frame from a. (Scale bars = 80 nm; n = 4 each group; PSD,postsynaptic density; SC,synaptic cleft; SV, synaptic vesicle)

The synaptic structure is the structural basis of synaptic transmission and synaptic plasticity. We observed the synaptic structure of each group of TNC neurons through TEM (Fig.5). In the sham group, the synapse structure was clear, the outline was complete, and many synaptic vesicles were seen. Compared with the sham group, the synaptic structure of the CM and the CM + DMSO groups was blurred, the synaptic cleft width, the thickness of PSD increased, and the synaptic interface curvature increased. The administration of MPEP restored the changes in the morphological indicators of synapses (Table 3).

**Table 3:** Synaptic ultrastructure parameters in the different groups

n = 4	Sham	CM	CM + DMSO	CM + MPEP
Thickness of the PSD/nm	21.33 ± 0.8536	37.24 ± 1.1464*	39.25 ± 1.2172*	19.74 ± 0.9326^#^
Width of the synaptic cleft/nm	19.29 ± 1.5273	32.13 ± 1.2319*	30.87 ± 2.0146*	20.92 ± 1.5236^#^
Active zones/nm	315 ± 24.58	496 ± 18.40*	507 ± 28.54*	342 ± 20.27^#^
Synaptic interface curvature	1.03 ± 0.0021	1.36 ± 0.02537*	1.39 ± 0.0725*	1.12 ± 0.02527^#^

*n* the number of rats. Date are presented as the mean ± SD. *p < 0.05 compared with the sham group; ^#^*p* < 0.05 compared with the CM + DMSO group

### MGluR5 regulated the number of neuronal dendritic spines

**Fig. 6:**
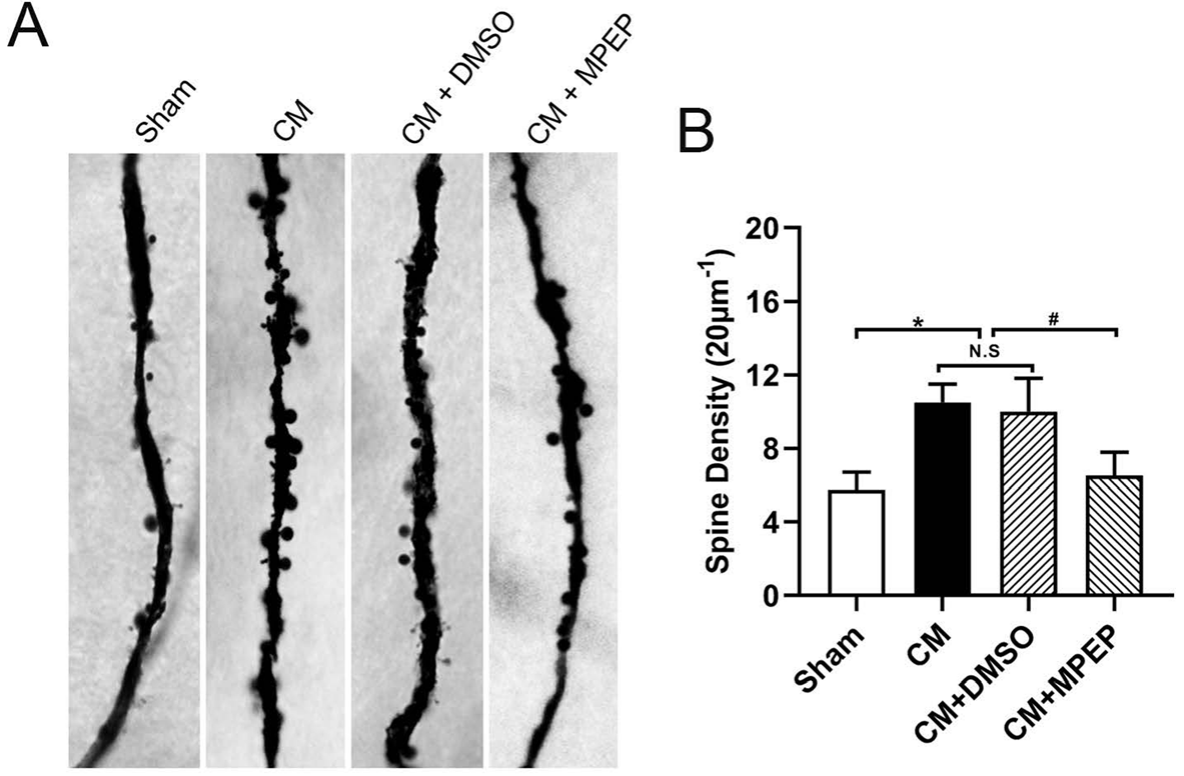
Dendritic spine density of TNC neurons in each group. **a** Representative photos of dendritic spines in each group. **b** Compared with the sham group, the density of dendritic spines in the CM group significantly increased, and there were no significant differences between the CM and CM + DMSO groups. The administration MPEP significantly reduced the density of dendritic spines. (n = 4 in each group,scale bar = 5 μm; *p < 0.05 compared with the sham group; ^#^p < 0.05 compared with the CM + DMSO group; N. S:p > 0.05 between two groups)

Dendritic spines, the main postsynaptic sites of excitatory synaptic input, were important components of synaptic function and plasticity [22, 23]. We detected the dendritic spines of pyramidal neurons in the TNC area by Golgi-Cox staining (Fig. 6a and b). Compared with the sham group, the density of dendritic spines in the CM group significantly increased, and no significant differences were found between the CM and CM + DMSO groups. The administration MPEP significantly reduced the density of dendritic spines. These results indicate that the dendritic spine density is increased in CM rats and is associated with mGluR5.

### MGluR5 regulated the PKC/NR2B signal in CM rats

**Fig. 7:**
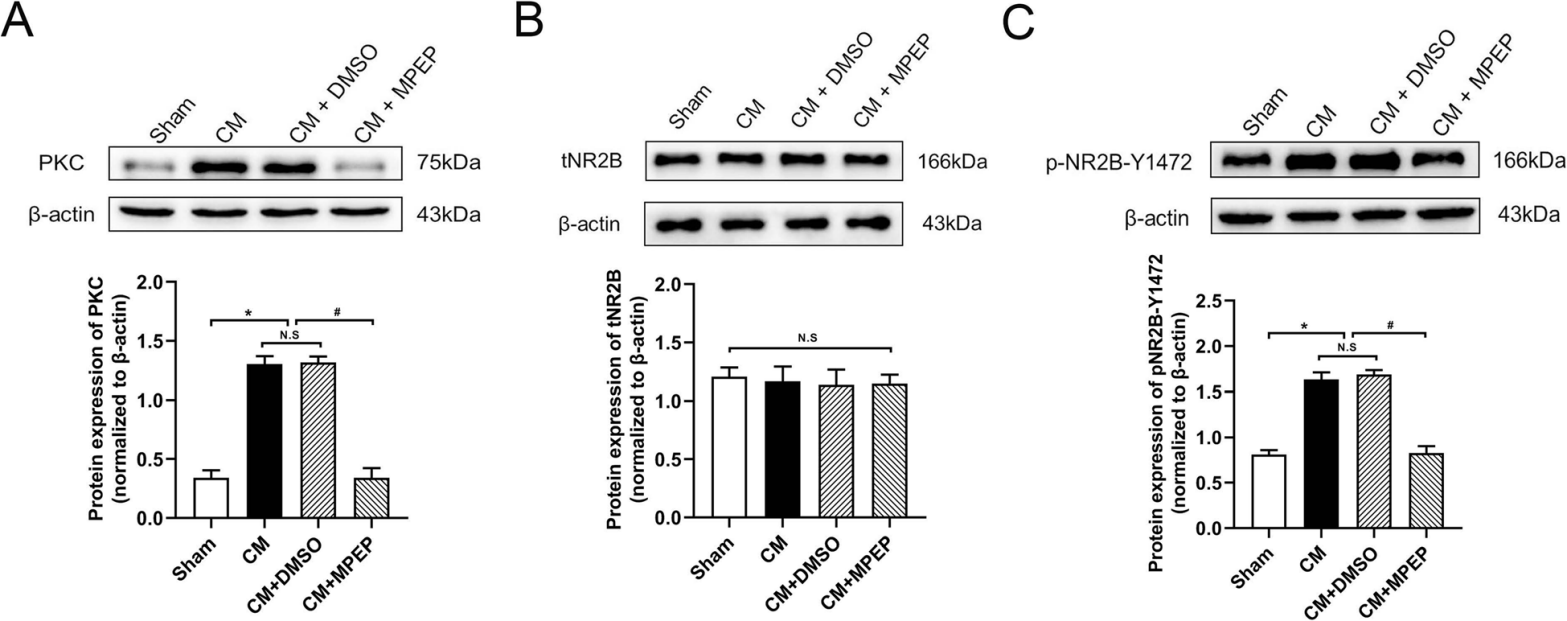
The effect of MPEP on the expression of PKC and phosphorylation of NR2B at Tyr1472. Compared with the sham group, the expression of PKC and phosphorylation of NR2B at Tyr1472 increased in the CM group. There were no significant differences between the CM and CM + DMSO groups. The administration of MPEP significantly downregulated their expression. **a** The protein expression of PKC. **b** The protein expression of total NR2B. **c** The protein expression of phosphorylation of NR2B at Tyr1472. (n = 4 in each group; *p < 0.05 compared with the sham group; ^#^p < 0.05 compared with the CM + DMSO group; N. S:p > 0.05 between two groups)

To investigate whether mGluR5 regulates the synaptic plasticity through PKC/NR2B signal in CM, we detected the protein expression of PKC, tNR2B, and pNR2B-Y1472. We found that the expression of PKC (Fig. 7a) and pNR2B-Y1472(Fig. 7c) significantly increased in the CM group, while the expression of tNR2B(Fig. 7b) had no significant difference. After the lateral ventricle administration of MPEP, the PKC and pNR2B-Y1472 expression decreased. These results support the notion that mGluR5 may regulate synaptic plasticity by PKC/NR2B signal.

### Downregulation of mGluR5 attenuated the central sensitization in CM rats

**Fig. 8:**
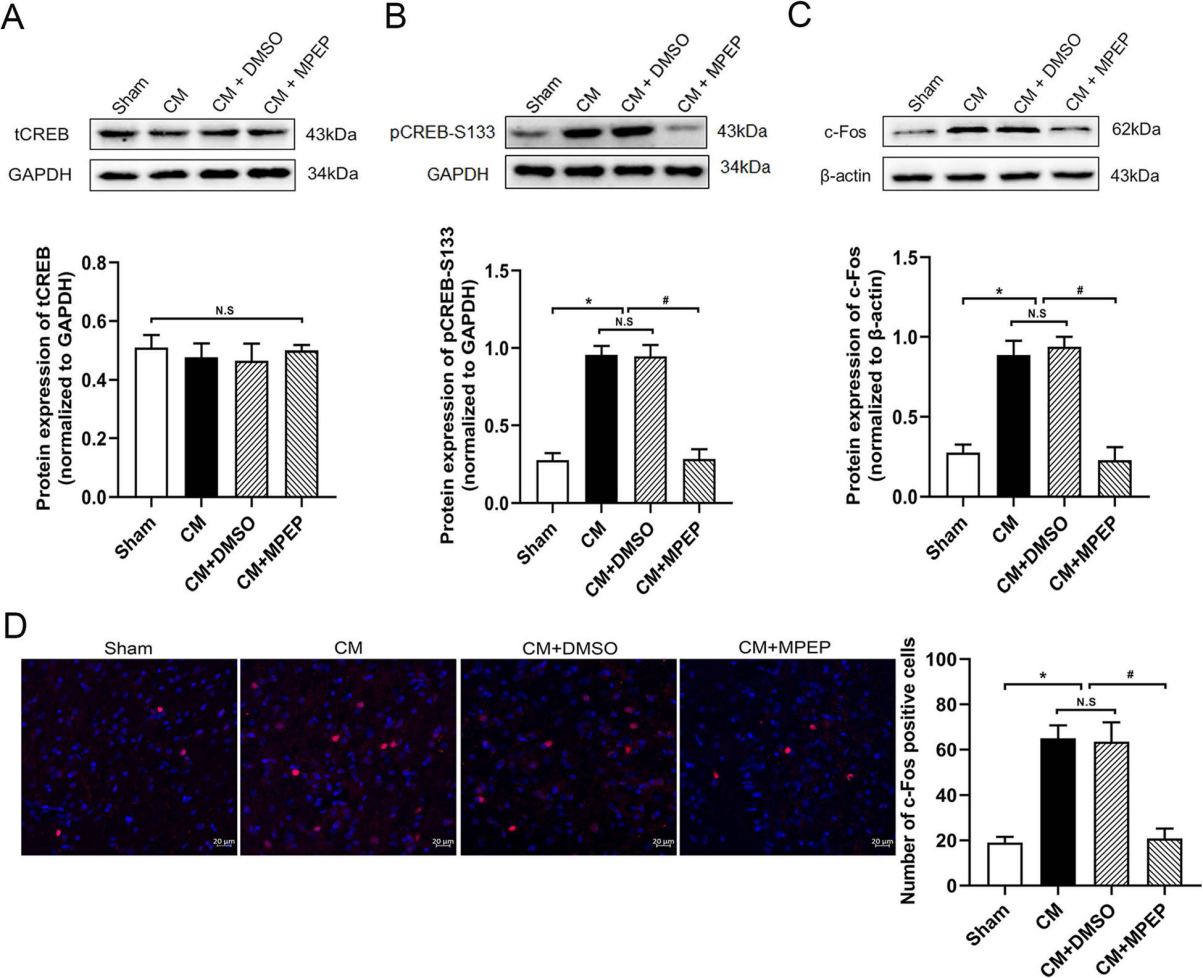
The effect of MPEP on the expression of p-CREB-S133 and c-Fos. Compared with the sham group, the expression of p-CREB-S133 and c-Fos increased in the CM group. There were no significant differences between the CM and CM + DMSO groups. The administration of MPEP significantly downregulated their expression. **a** The protein expression of CREB. **b** The protein expression of p-CREB-S133. **c** The protein expression of c-Fos. **d** Immunoreactivity for c-Fos. (n = 4 in each group, scale bar = 20 μm; *p < 0.05 compared with the sham group; ^#^p < 0.05 compared with the CM + DMSO group; N. S:p > 0.05 between two groups)

In order to explore whether mGluR5 is involved in the central sensitization of CM, we tested the expression of central sensitization-related indicators. CREB participates in the sensitization of noxious cells through phosphorylation. c-Fos is a classic marker of neuronal activation after noxious stimulation. We detected the expression of pCREB-S133 and c-Fos. The western blot results showed that the expression levels of pCREB-S133(Fig. 8b) and c-Fos (Fig. 8c) in the CM group were significantly higher than those in the sham group, and DMSO alone had no obvious effect, and the administration of MPEP reduced the expression of c-Fos and pCREB-S133. The expression of CREB (Fig. 8a) had no significant difference. The results of c-Fos immunofluorescence (Fig. 8d) were consistent with the expression of the c-Fos protein. The above results indicate that mGluR5 may regulate the central sensitization of CM.

## Discussion

The purpose of this study was to investigate whether mGluR5 in the TNC contributes to central sensitization of CM by regulating synaptic plasticity. First, the mRNA and protein expression levels of mGluR5 in the TNC were significantly increased in the CM model rats. Second, the treatment of MPEP, an antagonist of mGluR5, alleviated hyperalgesia induced by repeated dural infusion of IS and decreased the expression of CGRP, CREB-S133, and c-Fos. Third, the administration of MPEP restored synaptic structural changes and downregulated the expression of synaptic plasticity-related proteins PSD95, Syp, and Syt-1. Fourth, inhibition of mGluR5 decreased the high expression of PKC and phosphorylation of NR2B in CM. All these results suggest that mGluR5 may promote synaptic plasticity to contribute to the central sensitization of CM through PKC/NR2B signaling.

We used internationally recognized and reliable surgical catheterization and repeated infusion of IS to establish a rat model of CM [24]. To avoid the influence of estrogen fluctuations, male specific pathogen-free Wistar rats were selected for the experiment [25, 26]. Serotonin, histamine, bradykinin, and prostaglandin E2 continuously stimulate dural nociceptive receptors to activate the trigeminal neurovascular system. The significant decrease of mechanical pain thresholds and thermal pain thresholds were signs of the successful construction of the CM rat model [27]. CGRP is a neuropeptide that plays a vital role in the pathophysiology of migraine. The concentration of CGRP in the blood and cerebrospinal fluid of migraine patients increased. Besides, the gene expression of CGRP increased in the trigeminal ganglia and central nervous system of rats in the NTG-induced migraine model [28]. Therefore, biological indicator CGRP was also used to evaluate the model, after repeated infusion of IS into the dura mater for seven days, the expression of CGRP increased. All these are consistent with the clinical manifestations of CM patients [29], indicating that the CM rat model is successfully constructed.

Glutamate, the main excitatory amino acid, participates in the physiological transmission of nociceptive information and pain hypersensitivity. Studies have found that there are elevated glutamate levels in the blood, cerebrospinal fluid, occipital cortex, and thalamus of migraine patients [30]. MGluR5, the metabolic glutamate receptor, plays an important role in the nociceptive transmission of a variety of central nervous system diseases [31]. In our study, we found that there was an increase in the expression level of mGluR5 in CM. Intracerebroventricular administration of mGluR5 antagonist MPEP reversed the decrease of mechanical pain thresholds and thermal pain thresholds in CM rats induced by repeated dural infusion of IS, which was consistent with the results of previous studies. Blocking mGluR5 relieved complete Freund’s adjuvant (CFA) or carrageenan induced inflammatory hypersensitivity [32]. In addition to the inflammatory model, mGluR5 antagonist MPEP reversed mechanical hyperalgesia in spinal nerve ligation, sciatic nerve constriction, and vincristine induced neuropathic pain [33]. These show that mGluR5 plays a vital role in nociceptive perception and transmission in a variety of pain models, and our research has also confirmed its important role in CM.

CGRP plays an important role in the modulation of nociceptive transmission and central sensitization of CM. Our studies have confirmed the increased expression of CGRP in CM, the use of mGluR5 antagonist MPEP downregulated their expression in CM. In addition, we also detected the effect of MPEP on the expression of CREB and c-Fos. The phosphorylation of CREB at Serine 133 is considered to be an important indicator of the initiation and maintenance of central sensitization [34–36], and it can activate the downstream index c-Fos. C-Fos, an immediate early gene product, is a marker of neuronal activation [37, 38]. Our study confirmed the phosphorylation of CREB at Serine 133 and c-Fos were increased in CM rats. In addition, MPEP downregulated their expression. These findings suggest that mGluR5 contributes to neuronal activation and central sensitization in the CM rat model.

We confirmed that mGluR5 regulates the expression of central sensitization-related indicators in CM. Central sensitization refers to increased excitability of neurons in nociceptive pathways [39], so we speculated that mGluR5 may participate in the central sensitization by regulating the neural synaptic plasticity in CM. We examined the expression of several synaptic plasticity-related proteins. PSD-95 is the most abundant structural protein in postsynaptic densification in the central nervous system [40]. It can maintain and regulate synaptic plasticity by forming signal complexes with NMDA receptors or potassium channels, which plays an important role in synaptic efficiency transmission [41]. SYP is a special synaptic vesicle protein, which can regulate synaptic plasticity by affecting synaptic structure and neurotransmitter release. Its expression level can indirectly reflect the integrity and functional state of synaptic structure and is a specific marker of synaptic plasticity [42]. Syt-1 is a calcium-binding protein that dominates the release of calcium-dependent transmitters, participates in the discharge and transport of synaptic vesicles, affects synaptic function and structure, and is a marker of synaptic plasticity [43]. Similar to previous studies, our study found that the expression of these synaptic structures and function related proteins was significantly increased in CM, and the administration of MPEP downregulated their expression and regulated synaptic plasticity. In addition to the detection of synaptic plasticity-related proteins, we also examined the synaptic ultrastructure of TNC neurons in CM by TEM further confirmed mGluR5 involved in the regulation of synaptic plasticity in CM rats.

As one of the ionic receptors of glutamate, the NR2B receptor plays a vital role in pain and central sensitization in CM. Previous studies have shown that phosphorylation of NR2B at Tyr1472 plays a key role in synaptic plasticity [44]. Our study also confirmed that NR2B phosphorylation regulates synaptic plasticity in CM [7]. Intracerebroventricular injection of MPEP, an antagonist of mGluR5, decreased phosphorylation of NR2B at Tyr1472 in CM rats, suggesting that mGluR5 may be involved in the regulation of synaptic plasticity of CM by phosphorylating NR2B. Moreover, there were many studies on the relationship between mGluR5 and NMDA receptors in the regulation of synaptic plasticity. SH3 and multiple ankyrin repeat domains 3 (Shank3) proteins connect mGluR5 and NMDAR through PSD-95. The activation of mGluR5 enhances NMDAR currents in many cell types [45]. CA1 neurons in mGluR5-deficient mice showed the loss of NMDAR-mediated components of LTP [46], which indicates that mGluR5 can regulate NMDAR-mediated synaptic plasticity. In addition, NMDA-mediated LTP deficit in mGluR5-deficient mice was rescued by stimulating PKC [47]. Some studies have also shown that PKC participates in the synaptic plasticity of the central nervous system through NMDAR/CaMKII phosphorylate CREB [15, 16]. Similar to the previous results, we found that MPEP downregulated the increase of PKC and NR2B phosphorylation in CM rats. It is suggested that mGluR5 may regulate synaptic plasticity through PKC/NR2B in CM rats.

## Conclusion

In conclusion, our study demonstrated that mGluR5 contributed to the central sensitization of CM. The inhibition of mGluR5 alleviated the allodynia and central sensitization by regulating synaptic plasticity in CM. Furthermore, mGluR5 may regulated synaptic plasticity in CM by PKC/NR2B signaling. Therefore, mGluR5 may be a potential therapeutic candidate.

## Data Availability

Data can be made available upon request.

## Abbreviations

CGRP: Calcitonin gene-related peptide
CM: Chronic migraine
CREB: Cyclic adenosine monophosphate (cAMP) response element-binding protein
DMSO: Dimethyl sulfoxide
IS: Inflammatory soup
mGluR5: Metabotropic glutamate receptor 5
MPEP: 6-methyl-2-(phenylethynyl) pyridine hydrochloride
NMDA: N-methyl-D-aspartate
NR2B: N-methyl-D-aspartate receptor subtype 2B subunit
pCREB-S133: Phosphorylation of CREB at Serine 133
PKC: Protein kinase C
pNR2B-Y1472: Phosphorylation of NR2B at Tyr1472
PSD95: Postsynaptic density 95
Syp: Synaptophysin
Syt-1: Synaptotagmin1
TEM: Transmission electron microscope
TNC: Trigeminal nucleus caudalis
